# Natural hybridization between two butterfly bushes in Tibet: dominance of F_1_ hybrids promotes strong reproductive isolation

**DOI:** 10.1186/s12870-021-02909-7

**Published:** 2021-03-10

**Authors:** Rongli Liao, Weibang Sun, Yongpeng Ma

**Affiliations:** 1 Yunnan Key Laboratory for Integrative Conservation of Plant Species with Extremely Small Populations/ Key Laboratory for Plant Diversity and Biogeography of East Asia, Kunming Institute of Botany, Chinese Academy of Sciences, Kunming, 650201 Yunnan China; 2 University of Chinese Academy of Sciences, Beijing, 100049 China

**Keywords:** Butterfly bushes, *Buddleja*, Hybridization, F_1_-dominated hybrids, Reproductive isolation

## Abstract

**Background:**

It has been recognized that a certain amount of habitat disturbance is a facilitating factor for the occurrence of natural hybridization, yet to date we are unaware of any studies exploring hybridization and reproductive barriers in those plants preferentially occupying disturbed habitats. *Buddleja* plants (also called butterfly bush) generally do grow in disturbed habitats, and several species with hybrid origin have been proposed, based solely on morphological evidence.

**Results:**

In the present study, we test the hypothesis that *B. × wardii* is of natural hybridization origin in two sympatric populations of three taxa including *B. × wardii* and its parents (*B. alternifolia* and *B. crispa*) plus 4 referenced parental populations, using four nuclear genes and three chloroplast intergenic spacers, as well as with 10 morphological characters. Our results suggest that at both sites *B. × wardii* is likely to be a hybrid between *B. alternifolia* and *B. crispa*, and moreover, we confirm that most of the hybrids examined are F_1_s. That these plants are F_1_s is further supported by morphology, as no transgressive characters were detected. *B. crispa* was found to be the maternal parent in the Bahe (BH) population, from cpDNA evidence. However, in the Taji (TJ) population, the direction of hybridization was difficult to establish due to the shared cpDNA haplotypes between *B. alternifolia* and *B. crispa*, however we still predicted a similar unidirectional hybridization pattern due to results from cross-specific pollination treatments which supported the “SI × SC rule”.

**Conclusions:**

The presence of mainly F_1_ hybrids can successfully impede gene flow and thus maintain species boundaries in parental species in a typical distribution of *Buddleja*, i.e. in disturbed habitats.

**Supplementary Information:**

The online version contains supplementary material available at 10.1186/s12870-021-02909-7.

## Background

Natural hybridization in plants is a common phenomenon, and is thought to play an important role in plant diversity and speciation [1–4]. Many empirical studies have focused on the onset of natural hybridization and have examined issues including reproductive barriers [5, 6], backcrossing and introgression which are sometimes involved in transference of adaptation [7–9] or acceleration of extinction by genetic swamping [10–12]. A phenomenon previously assumed to be rare but recently found to be common, is the presence of hybrids consisting mainly of F_1_s [13–17], due to intrinsic incompatibilities including F_1_ hybrid sterility and/or inviability [4, 13, 18], and extrinsic selection, with other genotypes of hybrids being outcompeted due to strong habitat selection [13, 14, 19, 20]. A certain amount of habitat disturbance may be able to promote the occurrence of natural hybridization [21–23], as on the one hand it breaks the original existence of ecological isolation between species and promotes hybridization, and on the other hand it also provides suitable habitat for hybrids. However, to date we are unaware of any studies exploring hybridization and reproductive barriers in those plants preferentially occupying disturbed and/or newly alternated habitats.

Unlike later generation hybrids, that generally exhibit wide morphological variance because of genetic segregation, F_1_ hybrids from particular parental species tend to have similar morphologies due to the complete combination of parental genomes [15]. Because of this, F_1_s have been often inaccurately described as new species, especially by taxonomists concerned chiefly with morphology. An example of this is *Rhododendron agastum* from Yunnan, China, which has long be treated as a good species [24]. Recently, however, its hybrid origin has been confirmed, and population studies at the type locality suggest that most hybrids are F_1_s [13].

The genus *Buddleja* L. (Scrophulariaceae), comprises more than 100 species and is widely distributed throughout tropical, subtropical and temperate regions of the Americas, Africa and Asia [25, 26]. The Sino-Himalayan region is a center of diversity for this genus, with over 75% of the Asian *Buddleja* species occurring in this area [27, 28]. Moreover, most *Buddleja* species prefer to grow in disturbed habitats (e.g. sides of roads and on riverbanks), which is typical of pioneer plants [29–31], and some species (e.g. *B. davidii*) can become invasive if introduced to new environments [32–34]. Due to substantial overlaps in distribution and flowering periods, as well as shared pollinators, interspecific hybridization is assumed to be relatively common in the genus [25, 26, 29]. However, to date, only a single case of natural hybridization has been confirmed using molecular data [24].

**Fig. 1:**
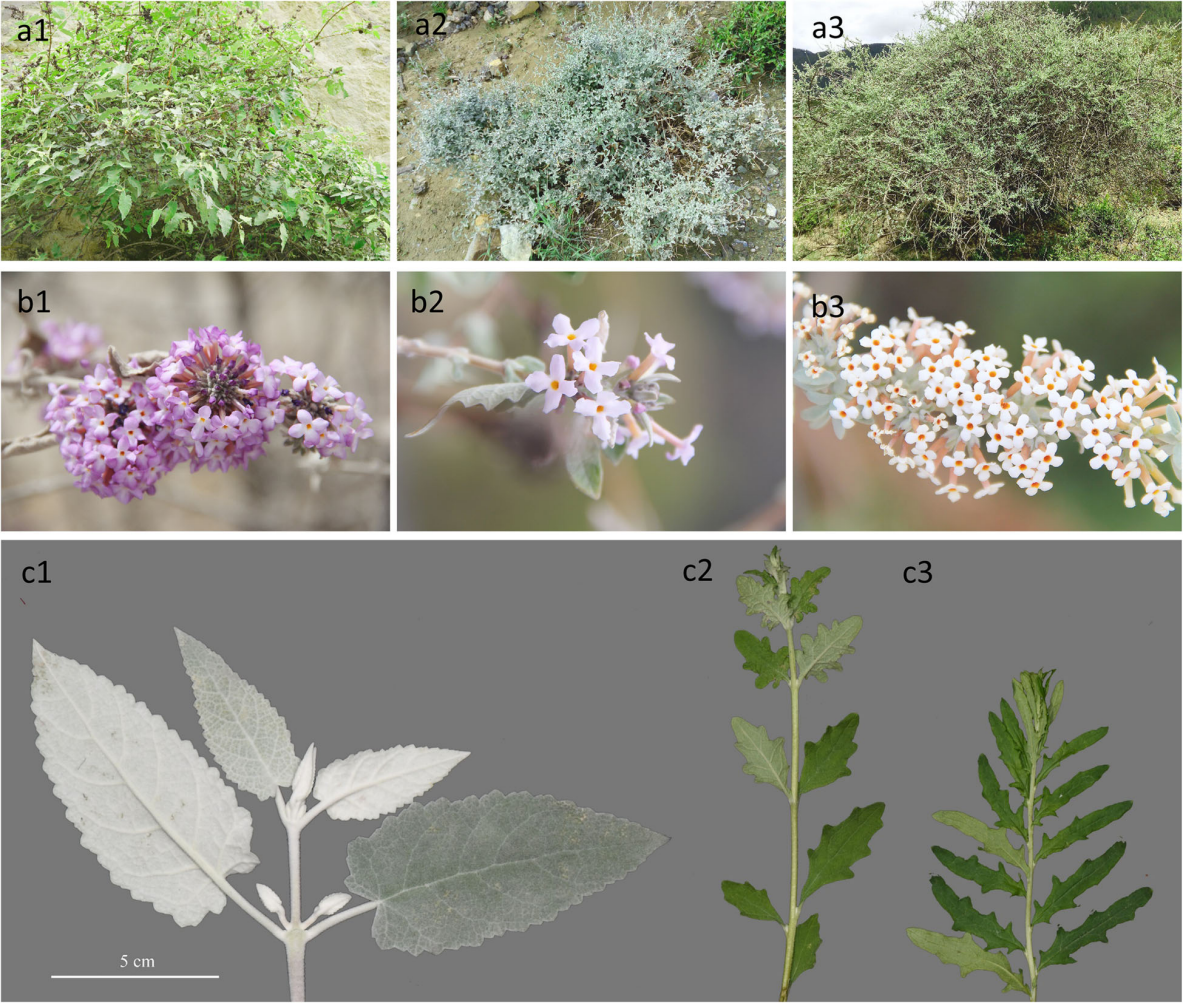
Morphological details of *B. crispa* (a1, b1, c1), *B. × wardii* (a2, b2, c2) and *B. alternifolia* (a3, b3, c3)

*Buddleja wardii* C. Marquand was originally described as a new species from Tibet by C. Marquand in 1929 [35]. Leeuwenberg [29] subsequently considered *B. wardii* to be a hybrid between the sympatric species *B. crispa* and *B. alternifolia*, based on the morphological characteristics of the type specimen and Ludlow c.s. 4098 (BM, E, K), as well as the fact that these two species are the only other *Buddleja* species found in the area. An important trait when determining *B. × wardii* plants is that the leaves are sometimes alternate and sometimes opposite on the same plant, even on the same stem (Fig. 1). *B. × wardii* is found in areas subject to frequent human activities, such as along artificial water channels or on the ridges between fields, where anthropogenic disturbance can be severe.

The aim of this study was to test the hypothesis that *B. × wardii* plants are natural hybrids, in two sites in Tibet. We used three chloroplast DNA regions and four nuclear genes used in previous studies on *Buddleja* (nr*ETS*, *gapC*2, *PPR*24, *PPR*123) [16, 36] to answer the following questions: 1) Are the *B. wardii* plants in fact hybrids between *B. crispa* and *B. alternifolia* at these two sites? 2) If yes, are there any differences between the genetic patterns at these two hybrid zones, both of which have serious habitat disturbance, and 3) does the genetic structure of hybrid zones such as these provide clues to the mechanism of reproductive isolation between parental species?

## Results

### Morphological analysis

Of the three leaf characters, leaf length (L), leaf width (W) and ratio of leaf length to leaf width (L/W), putative hybrid individuals consistently had two morphological characters, leaf length and leaf width, intermediate in value between *B. alternifolia* and *B. crispa* (Table 1). The L/W ratio was significantly larger in *B. alternifolia* than in the other two taxa (Table 1). Of the seven floral characters, corolla tube width (TW) and anther height (AH) in *B. × wardii* were intermediate between the values of the two assumed parental species, whereas herkogamy (HE) did not differ significantly between the three taxa (Table 1). The remaining four floral characters, corolla tube length (TL), corolla lobe length (CLL), corolla lobe width (CLW) and style length (SL) all showed a similar pattern, with the characters in *B. alternifolia* having significantly lower values than those measured in *B. crispa* or *B. × wardii* (Table 1).

**Table 1:** Morphological traits used to distinguish between *B. alternifolia*, *B. crispa* and *B. × wardii*

Characters	*B. alternifolia*	*B. × wardii*	*B. crispa*	F	Welch	*P* value
L (mm)	16.00 ± 3.15^a^	27.31 ± 10.02^b^	64.65 ± 25.47^c^		62.971	< 0.001
W (mm)	3.62 ± 0.66^a^	14.00 ± 3.26^b^	36.61 ± 11.72^c^		235.112	< 0.001
L/W	4.52 ± 0.99^a^	1.94 ± 0.47^b^	1.76 ± 0.31^b^		97.410	< 0.001
TL (mm)	7.10 ± 0.80^a^	9.77 ± 1.04^b^	10.25 ± 1.51^b^		79.584	0.017
TW (mm)	0.93 ± 0.17^a^	1.09 ± 0.15^b^	1.20 ± 0.18^c^	17.584		0.641
CLL (mm)	1.43 ± 0.27^a^	2.29 ± 0.37^b^	2.17 ± 0.40^b^	49.695		0.073
CLW (mm)	1.60 ± 0.20^a^	2.41 ± 0.35^b^	2.45 ± 0.46^b^		80.282	0.004
AH (mm)	5.22 ± 0.76^a^	5.97 ± 0.93^b^	6.66 ± 0.96^c^	18.266		0.539
SL (mm)	2.89 ± 0.47^a^	4.14 ± 0.91^b^	4.41 ± 1.05^b^		46.621	0.001
HE (mm)	1.08 ± 0.67	0.75 ± 0.72	0.95 ± 0.87	1.261		0.588

*1 L* leaf length, *W* leaf width, *L/W* ratio of leaf length to leaf width, *TL* corolla tube length, *TW* corolla tube width, *CLL* corolla lobe length, *CLW* corolla lobe width, *AH* anther height, *SL* style length, *HE* herkogamy

**Fig. 2:**
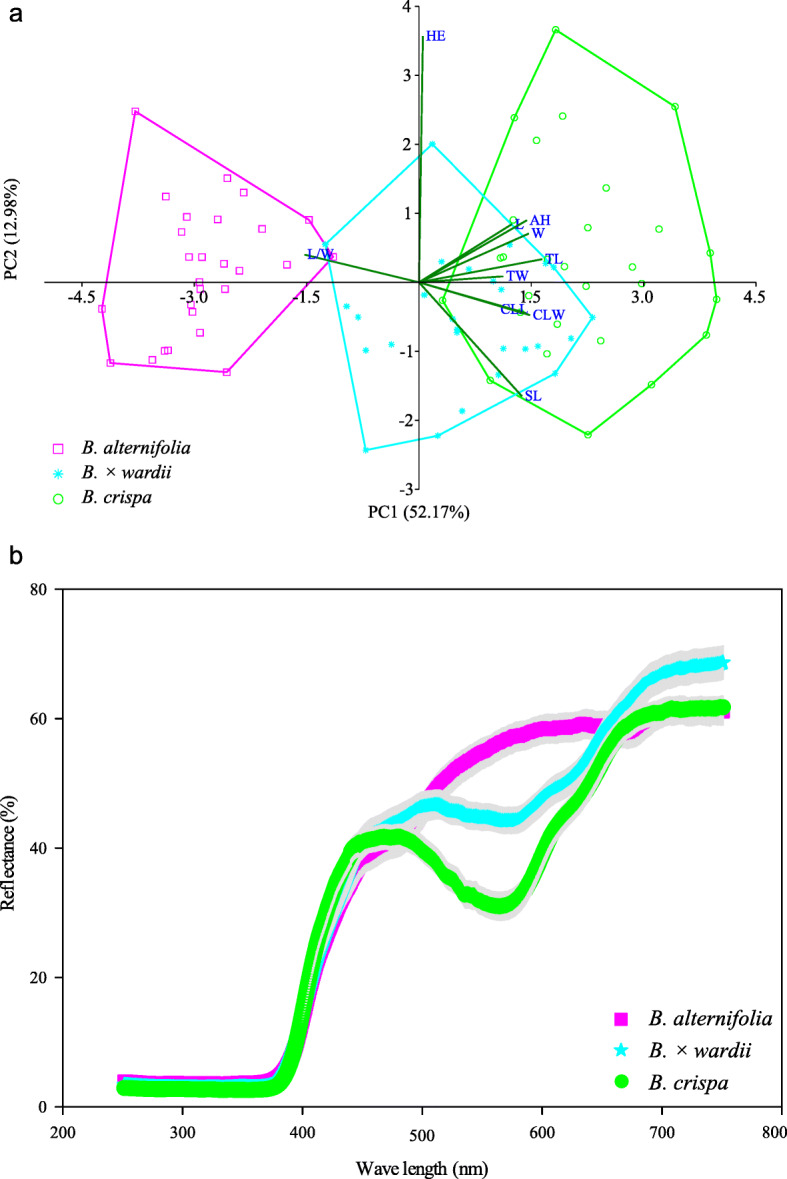
Two-dimensional scatter diagram of the first and second components from the PCA using 10 morphological characters (**a**), and petal reflectance spectra (**b**) in *B. alternifolia*, *B. crispa*, and *B. × wardii*. Pink squares, green circles and calamine blue stars represent *B. alternifolia*, *B. crispa* and *B. × wardii*, respectively

The two putative parental species are morphologically clearly distinct. In the PCA of 10 morphological characters, 52.17 and 12.98% of the variation in total was explained by the top two principal components, respectively. The two-dimensional scatter diagram based on PC1 and PC2 showed clearly the separation of *B. alternifolia* and *B. crispa*. Individuals of *B. × wardii* fell between the two parent species, with a slight overlap with *B. alternifolia* and a large overlap with *B. crispa*. Apart from the character HE, there is little difference in the correlation coefficients between the other nine traits (0.29–0.38) (Fig. 2a).

### Petal color reflectance

Differences in the reflectance spectrum of the corolla were found among the three study taxa. Both *B. crispa* and *B.* × *wardii* were found to have an obvious peak in the reflectance spectrum at 485 nm, with extremely low variation. However, there was no obvious peak in the reflectance spectrum in *B. alternifolia* (Fig. 2b).

### Pollination treatments

The two interspecific cross-pollination treatments, with either *B. crispa* or *B. alternifolia* as the mother species, showed significant differences in fruit set, mean number of seeds per fruit, seed set, and mean number of vigorous seeds per fruit. When *B. crispa* was the maternal plant, higher fruit set (64.71% vs 6.45%), higher numbers of seeds per capsule (43.42 ± 16.01 vs 6.5 ± 0.71), higher seed set (68.79% vs 0) and greater numbers of vigorous seeds per fruit (26.90 vs 0) were observed when comparing these parameters to cross-specific pollination with *B. alternifolia* as the maternal plant (Supplemental Table S1). Additionally, a comparatively high number of seeds was produced when *B. crispa* was subjected to geitonogamous pollination, indicating a self-compatible breeding system (seed set: 48.68%), whereas no seeds were obtained when *B. alternifolia* was subjected to hand self-pollination.

### Sequence analyses of the four nuclear genes

#### Nr*ETS*

**Fig. 3:**
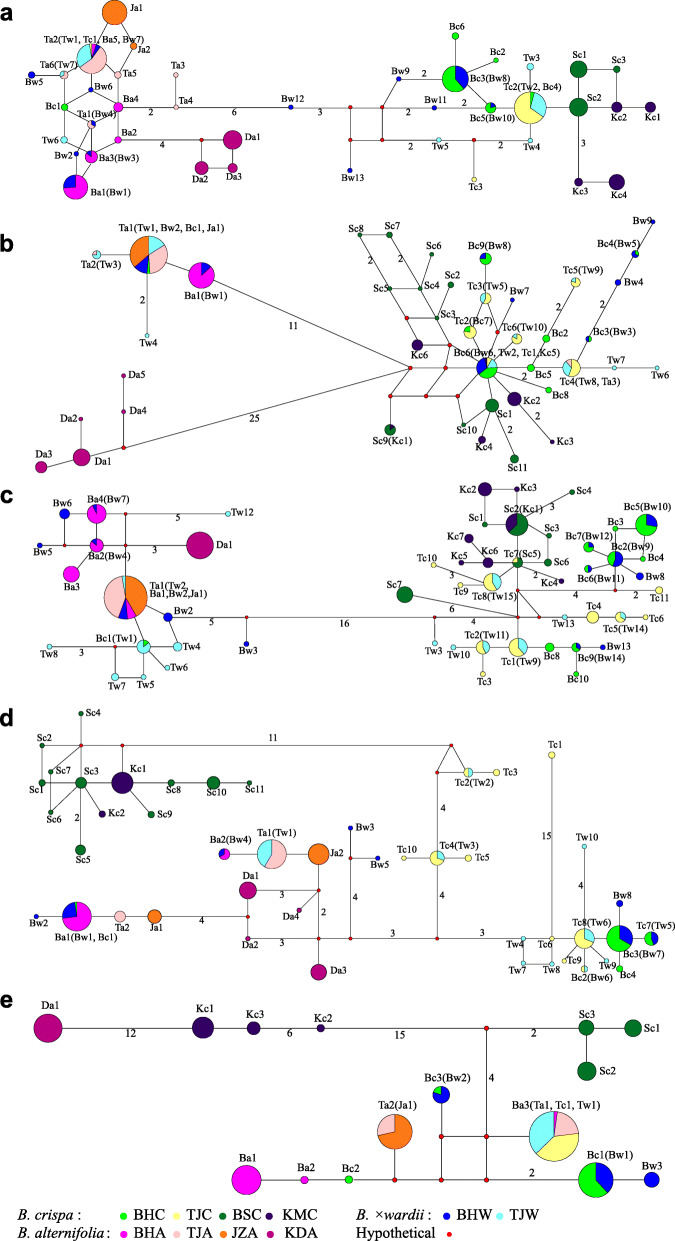
Haplotype network for *ETS* (**a**), *gapC*2 (**b**), * PPR*24 (**c**), *PPR*123 (**d**), cpDNA (**e**). Haplotypes from each taxon are denoted using the first letter of its population and species name (“Ba”, “Bc” and “Bw” refer to *B. alternifolia*, *B. crispa*, and *B.* × *wardii* fromt the BH population, respectively; while “Ta”, “Tc” and “Tw” refer to *B. alternifolia*, *B. crispa*, and *B.* × *wardii* from TJ population, respectively; “Da”, “Ja”, “Sc” and “Kc” refer to *B. alternifolia* from KD and JZ, and *B. crispa* from BS and KM, respectively. The number of mutations separating two adjacent haplotypes is shown by the number given on the connecting lines, the number is omitted for those with only one mutational step, and node size is proportional to the frequency of each haplotype. Colored circles represent haplotypes of different species as follows: green, yellow, dark green and dark blue represent *B. crispa*; pink, rose, orange and magenta represent *B. alternifolia*; blue and calamine blue represent *B. × wardii*. Small red circles represent hypothetical or unsampled haplotypes

The total length of the nr*ETS* region alignment was 380 bp in all individuals, including 30 nucleotide substitutions and one 1-bp insertion/deletion (for sites of variation, see Supplemental Table S2). These variable sites generated 40 haplotypes, of these, five (Ba), six (Bc) and thirteen (Bw) haplotypes were found in *B. alternifolia*, *B. crispa* and *B. × wardii* in the BH population, respectively, whereas six (Ta), three (Tc) and seven (Tw) haplotypes were found in the same species in the TJ population, respectively. For the four reference populations, samples from JZA, KDA, BSC and KMC represented two (Ja), three (Da), three (Sc) and four (Kc) specific haplotypes, respectively. Two major clusters were identified by haplotype network analysis with six nucleotide mutations. One cluster consisted mainly of haplotypes from *B. alternifolia* and *B.* × *wardii*, comprising five haplotypes from BHA, one haplotype from BHC, seven haplotypes from BHW, six haplotypes from TJA, one haplotype from TJC, three haplotypes from TJW and all haplotypes of *B. alternifolia* from the allopatric populations. The other cluster mainly consisted of haplotypes of *B. crispa* and *B.* × *wardii,* including five haplotypes of BHC, six haplotypes of BHW, two haplotypes of TJC, four haplotypes of TJW and all haplotypes of *B. crispa* from the allopatric populations (Fig. 3a).

Two haplotypes from *B. crispa* clustered together with the *B. alternifolia* cluster, and were found in BHCR2, BHCR7 and TJCR13, which show different haplotypes from both clusters. Except for two individuals, BHWI9 and TJWI1, all *B.* × *wardii* individuals with two haplotypes grouped into two divergent clusters, and mostly contained haplotypes from both *B. alternifolia* and *B. crispa* from the BH and TJ populations. The remaining two individuals had only one haplotype shared with *B. crispa* and were homozygous at this locus (Fig. 3a, Table 2).

#### *GapC*2

The total length of the *gapC*2 region alignment was 606 bp for all individuals, including 67 nucleotide substitutions, one 2-bp and one 1-bp insertion/deletion (for sites of variation, see Supplemental Table S2). A total of 41 haplotypes were observed from these loci of which one (Ba), nine (Bc) and nine (Bw) haplotypes belonged to *B. alternifolia*, *B. crispa* and *B. × wardii* in the BH population, whereas three (Ta), six (Tc) and ten (Tw) haplotypes belonged to these species in the TJ population*,* respectively. For the four reference populations, samples of JZA, KDA, BSC and KMC had one (Ja), five (Da), eleven (Sc) and four (Kc) specific haplotypes, respectively. The haplotype network analysis revealed three major clusters, with one cluster consisting of all haplotypes from KDA, the second cluster contained one haplotype from BHA, one haplotype from BHC, two haplotypes from BHW, two haplotypes from TJA, three haplotypes from TJW, and the only haplotype of JZA that was shared with the main haplotype from TJA. The third cluster contained eight haplotypes from BHC, seven haplotypes from BHW, six haplotypes from TJC, seven haplotypes from TJW and all haplotypes of *B. crispa* from the allopatric populations.

For *B. crispa* in both the TJ and BH populations, two individuals (BHCR2 and BHCR7) had a haplotype found in the second cluster, but all other haplotypes were found in the third cluster. For *B. alternifolia*, there was an exception of two individuals, TJAL6 and TJAL12, which had a haplotype found in the third cluster, but all the other haplotypes were found in the second cluster. All *B. × wardii* individuals but one (BHWI9) showed two divergent haplotypes originating from both the second and third clusters and mostly shared the haplotypes found in both *B. alternifolia* and *B. crispa* from the BH and TJ populations. The remaining individual, BHWI9, was homozygous for a haplotype from the third cluster (Fig. 3b, Table 2).

#### *PPR*24

The total length of the *PPR*24 region alignment was 647 bp for all individuals, including 70 nucleotide substitutions (for sites of variation, see Supplemental Table S2). This locus has the maximum number of haplotypes, 53 of all loci we studied. *B. alternifolia*, *B. crispa* and *B. × wardii* in the BH population had four (Ba), ten (Bc) and 14 (Bw) haplotypes, respectively, whereas there were one (Ta), 11 (Tc) and 15 (Tw) haplotypes in these species in the TJ population, respectively. For the four reference populations, samples from JZA, KDA, BSC and KMC had one (Ja), one (Da), seven (Sc) and seven (Kc) specific haplotypes, respectively. In the haplotypes network analysis, two major clusters were identified with 16 nucleotide substitutions. One cluster contained four haplotypes from BHA, one haplotype from BHC, seven haplotypes from BHW, the only haplotype from TJA, eight haplotypes from TJW and the unique haplotype for each of the two referenced populations of *B. alternifolia*. The other cluster contained nine haplotypes from BHC, seven haplotypes from BHW, all haplotypes from TJC, seven haplotypes from TJW, and all haplotypes of *B. crispa* from the allopatric populations. One *B. crispa* individual (BHCR7) and all *B. × wardii* individuals showed two divergent haplotypes originating from both clusters (Fig. 3c, Table 2).

#### *PPR*123

After sequence alignment, the *PPR*123 region was 735 bp in length for all samples including 59 nucleotide substitutions (for variation sites, Supplemental Table S2). A total of 45 haplotypes were identified for this gene, among which two (Ba), four (Bc) and eight (Bw) belong to *B. alternifolia*, *B. crispa* and *B. × wardii* in BH population, whereas two (Ta), ten (Tc) and ten (Tw) haplotypes in TJ population*,* respectively. For the four reference populations, samples of JZA, KDA, BSC and KMC had two (Ja), four (Da), eleven (Sc) and two (Kc) specific haplotypes, respectively. As shown in the haplotypes network analysis, all haplotypes were clustered into four groups with seven, six or 13 variations between them. The first clade comprised two haplotypes from BHA, one haplotype from BHC, five haplotypes from BHW, two haplotypes from TJA, one haplotype from TJA and all haplotypes of *B. alternifolia* from the allopatric populations. The second clade comprised three haplotypes from BHC, three haplotypes from BHW, five haplotypes from TJC and seven haplotypes from TJW. The third clade comprised five haplotypes from TJC and two haplotypes from TJW. The remaining clade comprised all haplotypes of *B. crispa* from the allopatric populations (Fig. 3d).

All haplotypes from *B. alternifolia* individuals fell into the first cluster. For the *B. crispa* individuals in BHC and TJC, only one from BHC clustered together with the first clade derived from BHCR7, ten individuals (TJCR3/5/6/7/9/10/11/12/13/15) had two divergent haplotypes, falling into the second and third clusters, four individuals (TJCR1/4/8/16) had haplotypes clustered in the second clade and two individuals (TJCR2/14) had haplotypes in the third clade. Of individuals of *B. × wardii*, all but BHWI9 had two different haplotypes, one from the first cluster and sharing the major haplotype with *B. alternifolia*, the other from either the second or the third cluster and sharing the major haplotypes with *B. crispa*. The remaining BHWI9 individual had two haplotypes found in the second clade (Fig. 3d, Table 2).

### Sequence analyses for the combined chloroplast regions

The combined aligned length of the cpDNA fragment alignment (including *rpl*16, *trn*D-*trn*T, *trn*S-*trnf*M) was 2014 bp, and contained 38 nucleotide substitutions, one 1-bp, one 2-bp and one 6-bp insertion/deletion (for sites of variation, see Supplemental Table S2). A total of 15 haplotypes were found across all samples, of which three (Kc), three (Sc) and one (Da) were specific to the reference populations of KMC, BSC and KDA, respectively. Haplotype network analysis indicated that these three reference populations differed from other populations by at least seven DNA base variants. All individuals from TJ had only two haplotypes, of which one was derived from six individuals from TJA (TJAL2/3/4/5/8/11) and shared with the only haplotype (Ja) from the reference population JZA, and the other haplotype, found in the remaining 42 individuals, was consistent with the haplotype found in individual BHAL13. Each taxon had three haplotypes in the BH population, among which two (Ba), one (Bc) and one (Bw) were specific to *B. alternifolia*, *B. crispa* and *B.* × *wardii*, respectively. Most of the individuals from BHW (75%) shared haplotypes with *B. crispa* (BHC)*,* while they did not share any haplotypes with *B. alternifolia*. The remaining four *B. × wardii* individuals (25%) had a unique haplotype one mutational step away from the predominant haplotype of *B. crispa* (Fig. 3e, Table 2).

### NewHybrids analysis

**Fig. 4:**
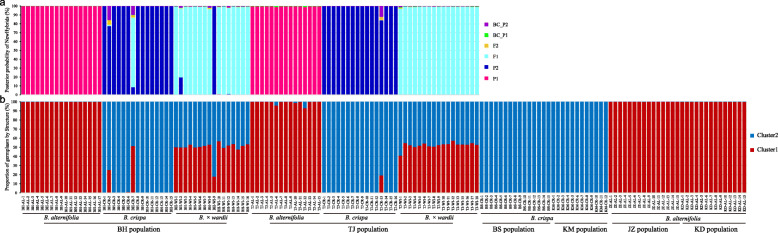
Genotype category assignment by NewHybrids (**a**) and Structure (**b**) for *B. alternifolia*, *B. crispa*, and *B. × wardii*. For the label of each accession, “BH”, “TJ”, “BS”, “KM”, “JZ” and “KD” refer to individuals collected in the populations BH, TJ, BS, KM, JZ and KD, respectively. “AL”, “WI” and “CR” refer to *B. alternifolia*, *B. crispa*, and *B. × wardii*, respectively*.* Bars in different color in **a** represent different genotype class: pink represents Parent I; Blue represents Parent II; calamine blue represents F_1_ hybrid; orange, red and purple represent F_2_ hybrid, backcross to Parent I and backcross to Parent II, respectively*.* Bars in different colors in **b** represent different clusters: blue and red represent Cluster 1 and Cluster 2, respectively

Analysis of the four studied nuclear genes using NewHybrids among the three taxa showed that individuals morphologically identified as *B. × wardii* from TJ and most individuals from BH were F_1_ hybrids between *B. alternifolia* and *B. crispa*. At BH, 31 of 33 individuals with putative parental morphology were assigned to the pure parental species with high posterior probabilities (> 98.8%). All individuals from BHW but two (BHWI2 and BHWI9) were identified as F_1_s with > 96.8% posterior probability. BHWI2 was identified as an F_1_ with a 77.8% posterior probability, while BHWI9 was identified as *B. crispa* with a 99.7% posterior probability (Fig. 4a).

At the TJ population, only TJCR13 was identified as *B. crispa*, with a low probability of 84.0%, and all the rest of the putative parental individuals were recognized as pure parents with high posterior probability (> 99.0%). All putative hybrids were identified as F_1_s with posterior probabilities of > 97.4% (Fig. 4a). Therefore, in both populations, three individuals were identified as backcrosses to *B. crispa* with a probability of less than 16%, which was far lower than their probability of being *B. crispa* or F_1_s, so the hybridization did not occur beyond the F_1_ generation.

### Population structure analysis

Structure analysis of a total of 213 variation loci for all individuals showed that the highest value of △K was obtained when K = 2, suggesting that all 152 individuals were clustered into two types of genetic clusters. When K = 2, for the four reference allopatric populations, all individuals from KMC and BSC formed a pure cluster (q ≥ 0.999), while all individuals from KDA and JZA formed another pure cluster (q ≥ 0.998).

As showed in the Fig. 4b, in the BH population, all individuals morphologically determined to be *B. alternifolia* clustered into one cluster with their high proportion containing one genetic component (q ≥ 0.997). Except for two individuals, BHCR2 and BHCR7, all individuals identified as *B. crispa* formed another cluster with the proportion containing another genetic component ≥0.998. While the proportion of the genetic component shared with individuals from population BHC was 0.755 in BHCR2 and 0.510 in BHCR7. All *B. × wardii* individuals showed approximately equal proportions from both clusters (q = 0.494 ± 0.084 for BHA), expect for BHWI9, which showed a proportion of 0.820 genetic component from the BHC cluster.

Similar to the BH population, most of the *B. alternifolia* (TJA) and *B. crispa* (TJC) from the TJ population contained a high proportion of different genetic components (q ≥ 0.988 for TJA; q ≥ 0.999 for TJC). Of the remaining three individuals, the probability that TJAL6 and TJAL12 contained low levels of genetic components from TJA was 0.959 and 0.931, respectively, while the probability that TJCR13 contained genetic components from TJC was 0.830. All individuals of *B. × wardii* (TJW) had similar proportions of genetic components from both TJA and TJC (q = 0.523 ± 0.033 for TJA).

## Discussion

In four of the quantitative morphological characters assessed, *B. × wardii* displayed characters that were intermediate between those of the parent species, whereas the remaining five characters were similar to those of *B. crispa* and different from those of *B. alternifolia.* No transparent traits were observed, indicating very early generation hybrids with limited genetic segregation. Chromatogram additivity of *B.* × *wardii* at each of the differentiated nuclear genes of the parental species (Table 2), as well as estimation of genotypes using NewHybrids further confirmed that most *B. × wardii* individuals should be considered F_1_s. Thus evidence from both morphology and molecular markers allowed us to reject the hypothesis that *B. × wardii* had undergone the sufficient genetic recombination assumed to be vital for establishing a diploid hybrid species [37]. Alternatively, F_1_s can successfully impede gene flow between parental species, and thus maintain species boundaries in *B. alternifolia* and *B. crispa* in sympatric areas. Intriguingly, we found that both parental species frequently occurred in disturbed habitats, and thus the empirical hypothesis of intermediate habitat, which has been deemed to be vital for hybrid establishment owing to the assumption of lower fitness of hybrids in both native habitats of parental species, was rejected [21].

**Table 2:** Haplotypes and genotypes of *B. × wardii* between *B. alternifolia* and *B. crispa* at four nuclear genes and the combined chloroplast regions (cpDNA). The code of each haplotype corresponds to those in Fig. 3

Individuals	*ETS*-B	*GapC*2	*PPR*24	*PPR*123	cpDNA
BHWI1	Ba1(Bw1)	Ba1(Bw1)	Bw6	Ba1(Bw1, Bc1)	Bc3(Bw2)
	Bc3(Bw8)	Bc4(Bw5)	Bc2(Bw9)	Bc3(Bw7)	
BHWI2	Bw2	Ta1(Tw1, Bw2, Bc1, Ja1)	Bw2	Bw5	Bc1(Bw1)
	Bc3(Bw8)	Bc6(Bw6, Tw2, Tc1, Kc5)	Bw13	Bc3(Bw7)	
BHWI3	Bw5	Ta1(Tw1, Bw2, Bc1, Ja1)	Bw6	Ba1(Bw1, Bc1)	Bc1(Bw1)
	Bc5(Bw10)	Bc9(Bw8)	Bc5(Bw10)	Bc2(Bw6)	
BHWI4	Bw5	Ta1(Tw1, Bw2, Bc1, Ja1)	Ba2(Bw4)	Ba1(Bw1, Bc1)	Bc1(Bw1)
	Bc3(Bw8)	Bc6(Bw6, Tw2, Tc1, Kc5)	Bc5(Bw10)	Bc3(Bw7)	
BHWI5	Ba1(Bw1)	Ba1(Bw1)	Bw6	Ba1(Bw1, Bc1)	Bc3(Bw2)
	Bc3(Bw8)	Bc4(Bw5)	Bc6(Bw11)	Bc3(Bw7)	
BHWI6	Ba3(Bw3)	Ba1(Bw1)	Bw5	Ba1(Bw1, Bc1)	Bc3(Bw2)
	Bc3(Bw8)	Bc6(Bw6, Tw2, Tc1, Kc5)	Bw8	Bw8	
BHWI7	Ba1(Bw1)	Ta1(Tw1, Bw2, Bc1, Ja1)	Bw6	Ba1(Bw1, Bc1)	Bc1(Bw1)
	Bw11	Bc6(Bw6, Tw2, Tc1, Kc5)	Bc2(Bw9)	Bc2(Bw6)	
BHWI8	Ba1(Bw1)	Ba1(Bw1)	Bw2	Ba2(Bw4)	Bw3
	Bw13	Bw7	Bc5(Bw10)	Bc3(Bw7)	
BHWI9	Bc3(Bw8)	Bw4	Bw3	Bc3(Bw7)	Bc3(Bw2)
	Bc3(Bw8)	Bw4	Bc2(Bw9)	Bw8	
BHWI10	Ba1(Bw1)	Ta1(Tw1, Bw2, Bc1, Ja1)	Bw2	Ba1(Bw1, Bc1)	Bc1(Bw1)
	Bw12	Bc3(Bw3)	Bw8	Bc2(Bw6)	
BHWI11	Ba1(Bw1)	Ta1(Tw1, Bw2, Bc1, Ja1)	Ba4(Bw7)	Ba2(Bw4)	Bw3
	Bc3(Bw8)	Bc6(Bw6, Tw2, Tc1, Kc5)	Bc5(Bw10)	Bc3(Bw7)	
BHWI12	Ta1(Bw4)	Ta1(Tw1, Bw2, Bc1, Ja1)	Ta1(Tw2, Ba1, Bw2, Ja1)	Bw2	Bc1(Bw1)
	Bc3(Bw8)	Bc6(Bw6, Tw2, Tc1, Kc5)	Bc7(Bw12)	Bc3(Bw7)	
BHWI13	Ta2(Tw1, Tc1, Ba5, Bw7)	Ta1(Tw1, Bw2, Bc1, Ja1)	Ta1(Tw2, Ba1, Bw2, Ja1)	Ba1(Bw1, Bc1)	Bc1(Bw1)
	Bc3(Bw8)	Bc9(Bw8)	Bc9(Bw14)	Bc3(Bw7)	
BHWI14	Ta2(Tw1, Tc1, Ba5, Bw7)	Ba1(Bw1)	Ta1(Tw2,Ba1, Bw2, Ja1)	Bw3	Bc1(Bw1)
	Bc3(Bw8)	Bw9	Bc2(Bw9)	Bc3(Bw7)	
BHWI15	Bw6	Ta1(Tw1, Bw2, Bc1, Ja1)	Ta1(Tw2, Ba1, Bw2, Ja1)	Ba1(Bw1, Bc1)	Bw3
	Bc3(Bw8)	Bc6(Bw6, Tw2, Tc1, Kc5)	Bc5(Bw10)	Bc3(Bw7)	
BHWI16	Ba1(Bw1)	Ta1(Tw1, Bw2, Bc1, Ja1)	Ta1(Tw2, Ba1, Bw2, Ja1)	Ba1(Bw1, Bc1)	Bw3
	Bw9	Bc6(Bw6, Tw2, Tc1, Kc5)	Bc2(Bw9)	Bc2(Bw6)	
TJWI1	Tw3	Ta2(Tw3)	Bc1(Tw1)	Ta1(Tw1)	Ba3(Ta1, Tc1, Tw1)
	Tw3	Tc3(Tw5)	Tc2(Tw11)	Tc7(Tw5)	
TJWI2	Tc2(Tw2, Bc4)	Ta2(Tw3)	Bc1(Tw1)	Ta1(Tw1)	Ba3(Ta1, Tc1, Tw1)
	Ta2(Tw1, Tc1, Ba5, Bw7)	Tc3(Tw5)	Tc1(Tw9)	Tw4	
TJWI3	Tc2(Tw2, Bc4)	Ta1(Tw1, Bw2, Bc1, Ja1)	Bc1(Tw1)	Ta1(Tw1)	Ba3(Ta1, Tc1, Tw1)
	Ta2(Tw1, Tc1, Ba5, Bw7)	Tw7	Tc2(Tw11)	Tw8	
TJWI4	Tc2(Tw2, Bc4)	Ta1(Tw1, Bw2, Bc1, Ja1)	Tw8	Ta1(Tw1)	Ba3(Ta1, Tc1, Tw1)
	Ta2(Tw1, Tc1, Ba5, Bw7)	Tw6	Tc2(Tw11)	Tw7	
TJWI5	Tc2(Tw2, Bc4)	Ta2(Tw3)	Bc1(Tw1)	Ta1(Tw1)	Ba3(Ta1, Tc1, Tw1)
	Ta2(Tw1, Tc1, Ba5, Bw7)	Tc4(Tw8, Ta3)	Tc8(Tw15)	Tw10	
TJWI6	Tc2(Tw2, Bc4)	Ta1(Tw1, Bw2, Bc1, Ja1)	Bc1(Tw1)	Ta1(Tw1)	Ba3(Ta1, Tc1, Tw1)
	Ta2(Tw1, Tc1, Ba5, Bw7)	Tc4(Tw8, Ta3)	Tc1(Tw9)	Tc4(Tw3)	
TJWI7	Tc2(Tw2, Bc4)	Ta1(Tw1, Bw2, Bc1, Ja1)	Tw7	Ta1(Tw1)	Ba3(Ta1, Tc1, Tw1)
	Ta2(Tw1, Tc1, Ba5, Bw7)	Tc6(Tw10)	Tc8(Tw15)	Tc7(Tw5)	
TJWI8	Tc2(Tw2, Bc4)	Tw4	Tw7	Ta1(Tw1)	Ba3(Ta1, Tc1, Tw1)
	Ta2(Tw1, Tc1, Ba5, Bw7)	Bc6(Bw6, Tw2, Tc1, Kc5)	Tc5(Tw14)	Tc7(Tw5)	
TJWI9	Tc2(Tw2, Bc4)	Ta1(Tw1, Bw2, Bc1, Ja1)	Bc1(Tw1)	Ta1(Tw1)	Ba3(Ta1, Tc1, Tw1)
	Ta2(Tw1, Tc1, Ba5, Bw7)	Tc4(Tw8, Ta3)	Tw13	Tc7(Tw5)	
TJWI10	Tc2(Tw2, Bc4)	Ta1(Tw1, Bw2, Bc1, Ja1)	Tw4	Ta1(Tw1)	Ba3(Ta1, Tc1, Tw1)
	Ta2(Tw1, Tc1, Ba5, Bw7)	Tc3(Tw5)	Tc1(Tw9)	Tc4(Tw3)	
TJWI11	Tc2(Tw2, Bc4)	Ta1(Tw1, Bw2, Bc1, Ja1)	Tw4	Ta1(Tw1)	Ba3(Ta1, Tc1, Tw1)
	Tw6	Bc6(Bw6, Tw2, Tc1, Kc5)	Tc1(Tw9)	Tc4(Tw3)	
TJWI12	Tw5	Ta1(Tw1, Bw2, Bc1, Ja1)	Tw4	Ta1(Tw1)	Ba3(Ta1, Tc1, Tw1)
	Ta2(Tw1, Tc1, Ba5, Bw7)	Tc5(Tw9)	Tc1(Tw9)	Tc2(Tw2)	
TJWI13	Tc2(Tw2, Bc4)	Ta1(Tw1, Bw2, Bc1, Ja1)	Tw6	Ta1(Tw1)	Ba3(Ta1, Tc1, Tw1)
	Tw6	Bc6(Bw6, Tw2, Tc1, Kc5)	Tc8(Tw15)	Tc2(Tw2)	
TJWI15	Tc2(Tw2, Bc4)	Ta1(Tw1, Bw2, Bc1, Ja1)	Ta1(Tw2, Ba1, Bw2, Ja1)	Ta1(Tw1)	Ba3(Ta1, Tc1, Tw1)
	Ta6(Tw7)	Tc4(Tw8, Ta3)	Tc8(Tw15)	Tc7(Tw5)	
TJWI16	Tc2(Tw2, Bc4)	Ta1(Tw1, Bw2, Bc1, Ja1)	Tw3	Ta1(Tw1)	Ba3(Ta1, Tc1, Tw1)
	Ta2(Tw1, Tc1, Ba5, Bw7)	Tc4(Tw8, Ta3)	Tw12	Tc7(Tw5)	
TJWI17	Tc2(Tw2, Bc4)	Ta1(Tw1, Bw2, Bc1, Ja1)	Ta1(Tw2, Ba1, Bw2, Ja1)	Ta1(Tw1)	Ba3(Ta1, Tc1, Tw1)
	Ta2(Tw1, Tc1, Ba5, Bw7)	Tc4(Tw8, Ta3)	Tw10	Tw9	
TJWI18	Tw4	Ta1(Tw1, Bw2, Bc1, Ja1)	Tw5	Ta1(Tw1)	Ba3(Ta1, Tc1, Tw1)
	Ta2(Tw1, Tc1, Ba5, Bw7)	Bc6(Bw6, Tw2, Tc1, Kc5)	Tc8(Tw15)	Tc8(Tw6)	

BHWI1-BHWI16 and TJWI1-TJWI13, 15-18 in the first column are 33 individuals of *B. × wardii* from the BH and TJ populations, respectively. “Ba”, “Bc” and “Bw” refer to *B. alternifolia*, *B. crispa*, and *B.* × *wardii* from the BH population, respectively; while “Ta”, “Tc” and “Tw” refer to *B. alternifolia*, *B. crispa*, and *B.* × *wardii* from the TJ population, respectively. In addition, “Kc” indicates *B. crispa* from KM and “Ja” indicates *B. alternifolia* from JZ

Most traditionally considered prerequisites for natural hybridization, i.e., sympatric distribution, overlapping flowering periods (*B. alternifolia* ranging from April to June, while *B. crispa* from March to August) [38, 39] and shared pollinators (bees, bumblebees and butterflies) [40] (personal observation) were fulfilled in *B. × wardii* [1], all facilitate natural hybridization between the two parental species. Another factor favoring natural hybridization between *B. alternifolia* and *B. crispa* was that they are both diploid with 2n = 38 [27], which may facilitate hybridization [41, 42]. In addition, *Buddleja* in Asia is a young clade that began diversifying approximately 10 Ma during the uplift of the Himalayas [27, 43], and reproductive isolation between them may be incomplete.

We never found isolated populations of *B. × wardii,* and in both of our study populations, it grows together with both putative parents. It is therefore probable that *B. × wardii* is not self sustainable [44, 45]. Taken together, our evidence suggests that *B. × wardii* should not be considered a hybrid species. We therefore recommended that the literature in the future should avoid the name *B. wardii*.

Although F_1_ dominant hybrid zones were confirmed in both study areas, heritable patterns of cpDNA were different. In most angiosperms, chloroplast DNA is maternally inherited [46]. The sequencing of cpDNA in the BH population showed that *B. crispa* and *B. alternifolia* each have their own specific chloroplast DNA haplotypes, and most *B.* × *wardii* individuals have the same haplotypes as *B. crispa*. The unique haplotypes seen in the remaining four *B.* × *wardii* individuals (BHWI8/11/15/16) may due to unsampled polymorphism of the parents. Several hypotheses have already been suggested to interpret asymmetric heritage of cpDNA in hybrids, including differences in breeding systems, flowering time, pollinator behavior and the local abundance of parental taxa in contact zone [5, 47–49]. For the present study, we have no evidence of pollinator behavior data to relate the asymmetry hybridization. It has been hypothesized that self-compatible species might act more commonly as the maternal parent than the self-incompatible species [13], which has been called the “SI × SC rule” [50]. Previous studies have confirmed self-compatibility in *B. crispa* from the substantial seed set attributed to geitonogamous pollination, similar to that following out-crossing [40], whereas no seeds were obtained following self-pollination treatments in *B. alternifolia*. Additionally, the peak flowering time in *B. crispa* is earlier than that in *B. alternifolia*, with only very limited overlap. Based on our observations of flowering time in individuals of the two parental species introduced into Kunming Botanical Garden from Tibet, *B. crispa* had nearly finished flowering when *B. alternifolia* started*.* Species with earlier flowering times are more likely to have more heterospecific pollen on the stigmas near the end of flowering due to the lack of homozygous pollen, and thus are more likely to be the maternal parent for hybrids [13, 51], assuming the shared pollinator(s) did not have visitation preference to one parent.

Additionally, in the TJ hybrid zone, the maternal parent could not be identified from the cpDNA results (only 6 individuals of *B. alternifolia* had a haplotype different from other individuals, with all other individuals of these three taxa sharing the same haplotype). Due to the fact that almost all hybrids were F_1_s and only a very limited number with low probabilities backcrossed to the parents (NewHybrids: *B. alternifolia*: 0; *B. crispa*: 1; Structure: *B. alternifolia*: 2; *B. crispa*: 1), the explanation that repeated backcrossing contributed to the pattern of cpDNA in this hybrid zone was rejected. We observed > 500 mature *B. alternifolia* and < 100 *B. crispa* individuals in TJ. The single haplotype of cpDNA might be explained by historical events (e.g. bottleneck) decreasing the haplotype diversity of cpDNA, which needs further study. Additionally, the identification of the maternal parent of any hybrids in a closely related species pair may be difficult, due to the slow evolution of the chloroplast genes [52, 53] or incomplete lineage sorting (ILS) of ancestral polymorphisms during rapid adaptive radiation in *Buddleja* [43]. However, as was discussed above, *B. crispa* could be still maternal plant of the hybrids in TJ due to the “SI × SC rule”.

## Conclusions

We investigated patterns of hybridization in two butterfly bush species in two locations in Tibet, both with elevations above 3400 m. Both morphological and molecular analyses supported the hypothesis that the putative hybrid plants are mainly F_1_s, which can further effectively promote nearly complete reproductive isolation between the parental plants. The self-compatible breeding system and earlier peak flowering period of *B. crispa* probably makes it the more frequent maternal parent, which is supported by the cpDNA analysis of hybrids. Overall, the present study provides insight into the maintenance of reproductive isolation, in particular for sympatrically growing pioneer plants in disturbed habitats, which have to date been largely ignored in natural hybridization studies.

## Methods

### Species and plant material in this study

Both *B. crispa* and *B. alternifolia* are vigorous deciduous shrubs or small trees to 2–4 m high. *B. crispa* is a widespread species found in the hot/warm-dry valleys, growing on forest edges, in shrubs, on exposed cliffs, and in rocks crevices at elevations of 1400–4300 m, across the Himalaya-Hengduan area [26, 54]. *B. alternifolia* is distributed in the northwest of China, throughout Tibet to the Loess Plateau, where it is naturally found growing along river banks or dried up streams in thickets at an altitude of 1500-4000 m [26, 55]. Ecologically, *B. crispa* and *B. alternifolia* are highly susceptible to habitat disturbance [29, 54, 55]. The two species occupy similar habitats and often occur sympatrically where their altitudinal ranges overlap [38, 54, 55]. Both are diploid with a chromosome number of 2n = 38 [27, 29, 56, 57], and start flowering in the spring (*B. crispa*: March to August; *B. alternifolia*: April to June) [38, 39].

**Fig. 5:**
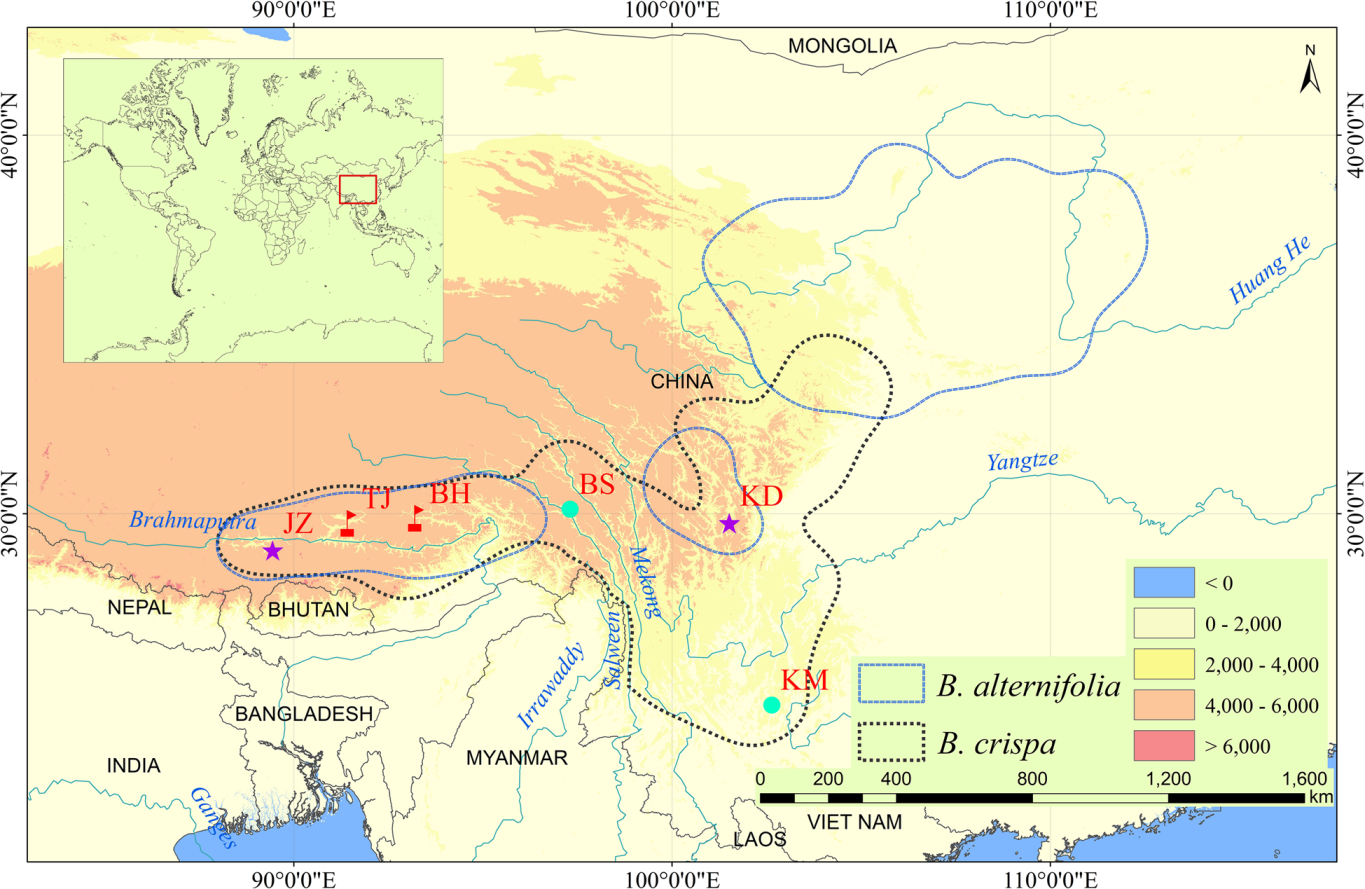
Known geographical distributions of *B. alternifolia* and *B. crispa* in China

All material for morphological characters and molecular analysis was field-collected. In Lhasa and Nyingchi, Tibet, China, individuals with morphologies intermediate between those of *B. alternifolia* and *B. crispa* were found co-occurring with sympatric populations of those two species along two branches of Brahmaputra river: the Ni-yang River and the Lhasa River (Fig. 5). In this study, we sampled 17, 15 and 16 individuals of *B. alternifolia*, *B. crispa* and *B. × wardii* from Bahe town (BH) in Nyingchi, and 15, 16 and 17 individuals of *B. alternifolia*, *B. crispa* and *B. × wardii* from Taji county (TJ) in Lhasa for molecular analysis. In addition, four allopatric populations of *B. alternifolia* and *B. crispa* were sampled as pure parental populations for reference: *B. alternifolia* from Jiangzi county in Rikaze, Tibet, and Kangding county in Tibetan Autonomous Prefecture of Garzê, Sichuan; *B. crispa* from Xishan Mountain in Kunming, Yunnan, and Basu county in Qamdo, Tibet. Sampling information is shown in Table 3. The Flora of China (FOC) was used for specific identification [38]. Voucher specimens were deposited at the herbarium of Kunming Institute of Botany, Chinese Academy of Sciences (Supplemental Table S3). In both sampled populations, many (more than 500) *B. alternifolia* individuals were found. However, at both BH and TJ, population size estimates of both *B. crispa* and the putative hybrid were fewer than 100 plants per population.

**Table 3:** Details of the sampling of *B. alternifolia*, *B. crispa* and *B. × wardii* in this study. The collection codes of the individual samples are given in brackets

Taxon	Sampling location	Number of individuals
*B. alternifolia*	Bahe, Nyingchi, Tibet, China (BHA)	17 (BHAL1–17)
	Taji, Lhasa, Tibet, China (TJA)	15 (TJAL1–15)
	Jiangzhi, Rikaze, Tibet, China (JZA)	15 (JZAL1–15)
	Kangding, Tibetan Autonomous Prefecture of Garzê, Sichuan, China (KDA)	14 (KDAL1–10,12–15)
*B. crispa*	Bahe, Nyingchi, Tibet, China (BHC)	15 (BHCR1–15)
	Taji, Lhasa, Tibet, China (TJC)	16 (TJCR1–16)
	Basu, Qamdo, Tibet, China (BSC)	15 (BSCR1–15)
	Xishan, Kunming, Yunnan, China (KMC)	12 (KMCR1–12)
*B. × wardii*	Bahe, Nyingchi, Tibet, China (BHW)	16 (BHWI1–16)
	Taji, Lhasa, Tibet, China (TJW)	17 (TJWI1–13,15–18)

### Measurements and data analysis of morphological traits

Thirty adult plants were randomly selected per taxon, and a fresh mature leaf from the middle of long, vegetative, current-growth branchlets without pests, diseases, or other obvious damage was sampled from each in June. Three leaf morphological characters were measured for each of these sampled leaves: 1) leaf length (L); 2) leaf width (W); 3) ratio of leaf length to leaf width (L/W). Seven morphological flower characters were measured from 30 randomly selected healthy flowers during the flowering period in April from 30 plants per taxon (one flower per individual): 4) corolla tube length (TL); 5) corolla tube width (TW, diameter of the top of corolla tube); 6) corolla lobe length (CLL); 7) corolla lobe width (CLW); 8) anther height (AH, from the top of stamen to the start of ovary); 9) style length (SL, from the top of stigma to the base of ovary); 10) herkogamy (HE, separation between tip of style and base of stamen). These 10 characteristics were measured using digital calipers to the nearest 0.01 mm (Supplemental Table S4).

A trimmed means was used to calculate the mean for the 30 individuals of each taxon [58]. One-way ANOVA was used to analyze these traits among the three taxa in SPSS Statistics 16.0 [59], and the significance of differences between the means was determined using the standard F statistic. Where the data did not satisfy the criterion of homogeneity of variance, a Welch statistic was employed. A post hoc Tamhane’s test was performed in pairwise comparison. The data were then subjected to two-dimensional Procrustes fitting in PAST (Palaeontology Statistical) software ver. 3.26 [60] to standardize landmark coordinates, followed by a shape principal components analysis (PCA) to perform multivariate analyses on the measured morphological characters (leaf and floral traits) [61].

### Petal color analysis

To assess patterns of light reflection in these three taxa at different wavelengths, a USB2000+ miniature fiber optic spectrometer with a DH2000 deuterium-halogen light source (Ocean Optics, Dunedin, FL, USA) was used to perform spectral measurements of the corolla color. Measurements were taken in increments of 0.45 and 0.55 nm over the range 250 nm to 750 nm [62]. We choose 30 petals (one flower from each of the 30 individuals per taxon) and took one measurement per petal (Supplemental Table S5).

### Artificial hybridization and seed germination

*B. alternifolia* plants from Tibet were successfully introduced in the Kunming Botanical Garden (KBG), and flowered in the spring of 2020. Artificial hybridization experiments were carried out on *B. alternifolia* and *B. crispa* growing in KBG, with these species as parents for three interspecific cross-pollination treatments (Supplemental Table S1). For all treatments, more than ten inflorescences with 1–3 single flowers and a total of 24, 31 and 34 single flowers were randomly selected, and were then, after artificial emasculation, hand-pollinated and bagged. Capsules were harvested just before they cracked and the number of fruits was counted. The number of seeds per fruit was counted after the capsules had cracked open naturally.

The collected seeds from the two interspecific pollination treatments were evenly sown in sterilized disposable Petri dishes with 9 cm diameter, lined with three pieces of filter paper and moistened with distilled water. A maximum of 50 seeds were sown per Petri dish and seeds were germinated at 20 °C for 12 h photoperiod in a plant growth chambers (MTI-202; Tokyo Rikakikai Co., Ltd., Japan) [63].

### DNA extraction, PCR amplification and DNA sequencing

Total DNA was extracted from approximately 50 mg dried leaves using the modified cetyl trimethyl ammonium (CTAB) method [64], and then stored at − 20 °C before further analyses. Three low-copy nuclear genes (*gapC*2, *PPR*24, *PPR*123), the nr*ETS* region, and three plastid regions (*rpl*16, *trn*D-*trn*T, *trn*S-*trnf*M) that had been successfully PCR amplified in *Buddleja* in previous publications were selected for sequencing [16, 36, 65]. Nr*ETS*, *gapC*2 and *PPR* are part of the nuclear ribosomal external transcribed spacer, the *gapC* gene family and the pentatricopeptide repeat gene family, respectively. Sequences of all the primers used are listed in Supplemental Table S6.

PCR was conducted using 2 × Taq PCR Master-mix (Tiangen) or the KOD-FX DNA polymerase (for *PPR*24). The PCR conditions were set as follows: initial denaturation at 94 °C for 4 min; then 32 cycles of 94 °C for 30 s, corresponding annealing temperature of 52 to 54 °C for 40 s, and 72 °C for 50 s; and an extension step at 72 °C for 10 min. After testing by running them on a 1.2% agarose gel, the PCR products were then sent for DNA sequencing on an ABI 3730 DNA analyzer (Applied Biosystems). Sequences have been deposited in GenBank with accession numbers MT7333350-MT733514 and MW591352-MW591467. In addition, sequence data for the *ETS* and *gapC*2 genes of 12 individuals from KMC were obtained from the paper published by Liao et al [16].

### Data analysis

The program SeqMan™ in DNASTAR was used for alignment, assembly, and comparison of DNA sequences [4, 16]. PHASE in DNASP ver. 5.10.01 was used to infer the haplotypes, and to calculate the number of haplotypes [66–68]. For the few haplotype estimates with uncertain phases, we choose the haplotype with the highest probability for analysis (Supplemental Table S7). Network ver. 5.0.1.1 was used to construct the haplotype network of each gene [61]. NewHybrids ver. 1.1 was used for the genotype class speculation of each individual: both parental species, F_1_s and F_2_s, backcrosses to each parental species [69] (Supplemental Table S8).

Genomic admixture proportions were determined using the program Structure ver. 2.3.4 with the default settings [70, 71]. Analyses were run with numbers of distinct clusters (K) varying from 1 to 15, with eight iterations performed for each K, a burn-in of 100,000 and a MCMC of 100,000 iterations (Supplemental Table S9). Structure Harvester web ver. 0.6.94 was used to obtain the optimal K of distinct groups [72, 73] (Supplemental Figure S1). The membership coefficients at each of the suggested numbers of clusters for each individual were estimated across the 8 independent runs and graphs of the population structuring were generated using Microsoft Excel 2016.

## Supplementary Information

**Additional file 1: Table S1.** Fruit set and seed set under different treatments in *B. alternifolia* and *B. crispa.*

**Additional file 2: Table S2.** Variable sites and indels of partial *ETS*, *gapC*2, *PPR*24, *PPR*123 genes and chloroplast intergenic spacer for haplotypes in all investigated individuals.

**Additional file 3: Table S3.** Specimens information for the three *Buddleja* taxa in the study.

**Additional file 4: Table S4.** 10 morphological trait data for the one-way ANOVA and principal components analyses.

**Additional file 5: Table S5.** Petal reflectance spectra in *B. alternifolia*, *B. crispa*, and *B. × wardii*.

**Additional file 6: Table S6.** Sequences of primers used in this study.

**Additional file 7: Table S7.** Haplotypes and genotypes of* B. alternifolia* and *B. crispa *at four nuclear genes and the combined chloroplast regions (cpDNA).

**Additional file 8: Table S8.** The probabilities of six genetic clusters from the NewHybrids analysis.

**Additional file 9: Table S9.** The probabilities of two genetic clusters from the Structure analysis.

**Additional file 10: Figure S1.** ΔK values for the Structure analysis.

## Data Availability

The data sets supporting the results of the present study are included within this article and its additional files.

## Abbreviations

ANOVA: Analysis of variance
DNA: DeoxyriboNucleic Acid
cpDNA: chloroplast DNA
PCR: Polymerase chain reaction
MCMC: Markov Chain Monte Carlo

## References

[1] Arnold ML. Natural hybridization and evolution. New York: Oxford University Press; 1997.

[2] Rieseberg LH (1995). The role of hybridization in evolution – old wine in new skins. Am J Bot.

[3] Soltis PS, Soltis DE (2009). The role of hybridization in plant speciation. Annu Rev Plant Biol.

[4] Zhang RS, Liu T, Wu W, Li YQ, Chao LF, Huang LS, Shi SH, Zhou RC (2013). Molecular evidence for natural hybridization in the mangrove fern genus *Acrostichum*. BMC Plant Biol.

[5] Zhou RC, Gong X, Boufford D, Wu CI, Shi SH (2008). Testing a hypothesis of unidirectional hybridization in plants: observations on *Sonneratia, Bruguiera* and *Ligularia*. BMC Evol Biol.

[6] Wei YK, Huang YB, Li GB (2017). Reproductive isolation in sympatric *Salvia* species sharing a sole pollinator. Biodivers Sci.

[7] Arnold ML (1993). (1993) *Iris nelsonii*: origin and genetic composition of a homoploid hybrid species. Am J Bot.

[8] Yan LJ, Gao LM, Li DZ (2013). Molecular evidence for natural hybridization between *Rhododendron spiciferum* and *R. spinuliferum* (Ericaceae). J Syst Evol.

[9] Zhang W, Dasmahapatra KK, Mallet J, Moreira GR, Kronforst MR (2016). Genome-wide introgression among distantly related *Heliconius* butterfly species. Genome Biol.

[10] Tagane S, Hiramatsu M, Okubo H (2008). Hybridization and asymmetric introgression between *Rhododendron eriocarpum* and *R. indicum* on Yakushima Island, Southwest Japan. J Plant Res.

[11] Todesco M, Pascual MA, Owens GL, Ostevik KL, Moyers BT, Hübner S, Heredia SM, Hahn MA, Caseys C, Bock DG, Rieseberg LH (2016). Hybridization and extinction. Evol Appl.

[12] Wu RZ, Zou PS, Tan GW, Hu ZY, Wang YQ, Ning ZL, Wu W, Liu Y, He SY, Zhou RC (2019). Molecular identification of natural hybridization between *Melastoma malabathricum* and *Melastoma beccarianum* in Sarawak, Malaysia. Ecol Evol.

[13] Zha HG, Milne RI, Sun H (2010). Asymmetric hybridization in *Rhododendron agastum*: a hybrid taxon comprising mainly F_1_s in Yunnan, China. Ann Bot.

[14] Milne RI, Terzioglu S, Abbott R (2003). A hybrid zone dominated by fertile F_1_s: maintenance of species barriers in *Rhododendron*. Mol Ecol.

[15] Milne RI, Abbott RJ (2008). Reproductive isolation among two interfertile *Rhododendron* species: low frequency of post-F_1_ hybrid genotypes in alpine hybrid zones. Mol Ecol.

[16] Liao RL, Ma YP, Gong WC, Chen G, Sun WB, Zhou RC, Marczewski T (2015). Natural hybridization and asymmetric introgression at the distribution margin of two *Buddleja* species with a large overlap. BMC Plant Biol.

[17] Zhang NN, Ma YP, Folk RA, Yu JJ, Pan YZ, Gong X (2018). Maintenance of species boundaries in three sympatric *Ligularia* (Senecioneae, Asteraceae) species. J Integr Plant Biol.

[18] Zhang NN, Yu JJ, Wang YH, Gong X (2018). Molecular evidence for asymmetric hybridization in three closely related sympatric species. AoB Plants.

[19] Kyhos DW, Clark C, Thompson WC (1981). The hybrid nature of *Encelia laciniata* (Compositae, Heliantheae) and control of population composition by post-dispersal selection. Syst Bot.

[20] Nagano Y, Hirao AS, Itino T (2015). Genetic structure of a hybrid zone between two violets, *Viola rossii* Hemsl. and *V. bissetii* maxim.: dominance of F_1_ individuals in a narrow contact range. Plant Spec Biol.

[21] Anderson E (1948). Hybridization of the habitat. Evolution..

[22] Grant PR, Grant BR (2014). Speciation undone. Nature..

[23] Mallet J (2005). Hybridization as an invasion of the genome. Trends Ecol Evol.

[24] Fang MY, Fang RZ, He MY, Hu LZ, Yang HB, Chamberlain DF, Wu ZY, Raven PH (2005). Rhododendron. Flora of China, Vol. 14.

[25] Norman EM (2000). Buddlejaceae.

[26] Stuart DD (2006). Buddlejas.

[27] Chen G, Sun WB, Sun H (2007). Ploidy variation in *Buddleja* (Buddlejaceae) in the sino-himalayan region and its biogeographical implications. Bot J Linn Soc.

[28] Ge J, Cai L, Bi GQ, Chen G, Sun WB (2018). Characterization of the complete chloroplast genomes of *Buddleja colvilei* and *B. sessilifolia*: Implications for the taxonomy of *Buddleja*. Molecules.

[29] Leeuwenberg AJM (1979). The Loganiaceae of Africa XVIII. *Buddleja* II. Revision of the African and Asiatic species.

[30] Gong WC, Chen G, Liu CQ, Dunn BL, Sun WB (2014). Comparison of floral scent between and within *Buddleja fallowiana* and *Buddleja officinalis* (Scrophulariaceae). Biochem Syst Ecol.

[31] Raja S, Ramya I (2016). A review on ethnopharmacology, phytochemistry and pharmacology of *Buddleja asiatica*. Int J Pharm Sci Res.

[32] Sheppard AW, Shaw RH, Sforza R (2006). Top 20 environmental weeds for classical biological control in Europe: a review of opportunities, regulations and other barriers to adoption. Weed Res.

[33] Tallent-Halsell NG, Watt MS (2009). The invasive *Buddleja davidii* (butterfly bush). Bot Rev.

[34] Wittig R (2012). Frequency of *Buddleja davidii* Franch. (Buddlejaceae) in Germany along ecological gradients. Flora..

[35] Marquand CVB (1929). The botanical collection made by captain F. Kingdon Ward in the eastern Himalaya and Tibet in 1924–25. Bot J Linn Soc.

[36] Chau JH, O'Leary N, Sun WB, Olmstead RG (2017). Phylogenetic relationships in tribe Buddlejeae (Scrophulariaceae) based on multiple nuclear and plastid markers. Bot J Linn Soc.

[37] Ma YP, Zhang CQ, Zhang JL, Yang JB (2010). Natural hybridization between *Rhododendron delavayi* and *R. cyanocarpum* (Ericaceae), from morphological, molecular and reproductive evidence. J Integr Plant Biol.

[38] Li PT, Leeuwenberg AJM, Wu ZY, Raven PH (1996). Loganiaceae. Flora of China, Vol. 15.

[39] Wang HY, Zhang XS, Li XQ, Zhao HH, Shi XB (2010). Rapid propagation of *Buddleja alternifolia*. Guizhou Agri Sci.

[40] Gong WC, Chen G, Vereecken NJ, Dunn BL, Ma YP, Sun WB (2014). Floral scent composition predicts bee pollination system in five butterfly bush (*Buddleja*, Scrophulariaceae) species. Plant Biol.

[41] Kou YX, Xiao K, Lai XR, Wang YJ, Zhang ZY (2017). Natural hybridization between *Torreya jackii* and *T. grandis* (Taxaceae) in Southeast China. J Syst Evol.

[42] Ning H, Pan YZ, Gong X (2019). Molecular evidence for natural hybridization between *Ligularia nelumbifolia* and *Cremanthodium stenoglossum* (Asteraceae, Senecioneae). Botany.

[43] Chau JH (2017). Systematics of *Buddleja* (Scrophulariaceae): phylogenetic relationships, historical biogeography, and phylogenomics.

[44] Abbott RJ (2003). Sex, sunflowers, and speciation. Science..

[45] Feliner GN, Alvarez I, Fuertes-Aguilar J, Heuertz M, Marques I, Moharrek F, Pineiro R, Riina R, Rossello JA, Soltis PS, Villa-Machio I (2017). Is homoploid hybrid speciation that rare? An empiricist's view. Heredity..

[46] Morgensen HL (1996). The hows and whys of cytoplasmic inheritance in seed plants. Am J Bot.

[47] Ma YP, Milne R, Zhang CQ, Yang JB (2010). Unusual patterns of hybridization involving a narrow endemic *Rhododendron* species (Ericaceae) in Yunnan, China. Am J Bot.

[48] Ma YP, Xie WJ, Tian XL, Sun WB, Wu ZK, Milne R (2014). Unidirectional hybridization and reproductive barriers between two heterostylous primrose species in north-West Yunnan, China. Ann Bot..

[49] Tong ZY, Huang SQ (2016). Pre- and post-pollination interaction between six co-flowering *Pedicularis* species via heterospecific pollen transfer. New Phytol.

[50] Lewis D, Crowe LK (1958). Unilateral interspecific incompatibility in flowering plants. Heredity.

[51] Zhang JJ, Montgomery BR, Huang SQ (2016). Evidence for asymmetrical hybridization despite pre- and post-pollination reproductive barriers between two *Silene* species. AoB Plants.

[52] Wolfe KH, Li WH, Sharp PM (1987). Rates of nucleotide substitution vary greatly among plant mitochondrial, chloroplast, and nuclear DNAs. Proc Natl Acad Sci.

[53] Drouin G, Daoud H, Xia J (2008). Relative rates of synonymous substitutions in the mitochondrial, chloroplast and nuclear genomes of seed plants. Mol Phylogenet Evol.

[54] Yue LL, Chen G, Sun WB, Sun H (2012). Phylogeography of *Buddleja crispa* (Buddlejaceae) and its correlation with drainage system evolution in southwestern China. Am J Bot.

[55] Wang JH, Zhang JG, Xu Y, Wu HW, Sun GP (2007). Study on biology characteristics of drought enduring shrub *Buddleja alternifolia*. Chinese Wild Plant Resour.

[56] Moore RJ (1947). Cytotaxonomic studies in the Loganiaceae I. chromosome numbers and phylogeny in the Loganaceae. Am J Bot.

[57] Moore R (1961). Polyploidy, phylogeny, and photoperiodism in Old World *Buddleja*. Evolution..

[58] Marczewski T, Ma YP, Zhang XM, Sun WB, Marczewski AJ (2016). Why is population information crucial for taxonomy? A case study involving a hybrid swarm and related varieties. AoB Plants.

[59] SPSS (2007). SPSS Statistics 16.0.

[60] Hammer Ø, Harper DAT, Ryan PD (2001). Past: paleontological statistics software package for education and data analysis. Palaeontol Electron.

[61] Bandelt HJ, Forster P, Röhl A (1999). Median-joining networks for inferring intraspecific phylogenies. Mol Biol Evol.

[62] Ma YP, Wu ZK, Dong K, Sun WB, Marczewski T (2015). Pollination biology of *Rhododendron cyanocarpum* (Ericaceae): an alpine species endemic to NW Yunnan, China. J Syst Evol.

[63] Sun WB, Kong FC, Mickael LW (2002). Effect of light and temperature on seed germination of *Buddleja crispa*. Plant Physiol Commun.

[64] Doyle JJ, Doyle JL (1987). A rapid DNA isolation procedure for small quantities of fresh leaf tissue. Phytochem Bulletin.

[65] Borg AJ, McDade LA, Schönenberger J (2008). Molecular phylogenetics and morphological evolution of Thunbergioideae (Acanthaceae). Taxon..

[66] Librado P, Rozas J (2009). Dnasp v5: a software for comprehensive analysis of DNA polymorphism data. Bioinformatics..

[67] Stephens M, Smith NJ, Donnelly P (2001). A new statistical method for haplotype reconstruction from population data. Am J Hum Genet.

[68] Stephens M, Scheet P (2005). Accounting for decay of linkage disequilibrium in haplotype inference and missing-data imputation. Am J Hum Genet.

[69] Anderson EC, Thompson EA (2002). A model-based method for identifying species hybrids using multilocus genetic data. Genetics..

[70] Hubisz MJ, Falush D, Stephens M, Pritchard JK (2009). Inferring weak population structure with the assistance of sample group information. Mol Ecol Resour.

[71] Pritchard JK, Stephens M, Donnelly P (2000). Inference of population structure using multilocus genotype data. Genetics..

[72] Earl DA, Vonholdt BM (2012). Structure harvester: a website and program for visualizing structure output and implementing the evanno method. Conserv Genet Resour.

[73] Evanno G, Regnaut S, Goudet J (2005). Detecting the number of clusters of individuals using the software STRUCTURE: a simulation study. Mol Ecol.

