# Supplementary figures and images for: Natural hybridization between two butterfly bushes in Tibet: dominance of F1 hybrids promotes strong reproductive isolation
Source: BMC Plant Biol. 2021 Mar 10;21:133. doi: 10.1186/s12870-021-02909-7 (PMC7945306; doi:10.1186/s12870-021-02909-7)

$$\text{DeltaK} = \text{mean}(|L''(K)|) / \text{sd}(L(K))$$

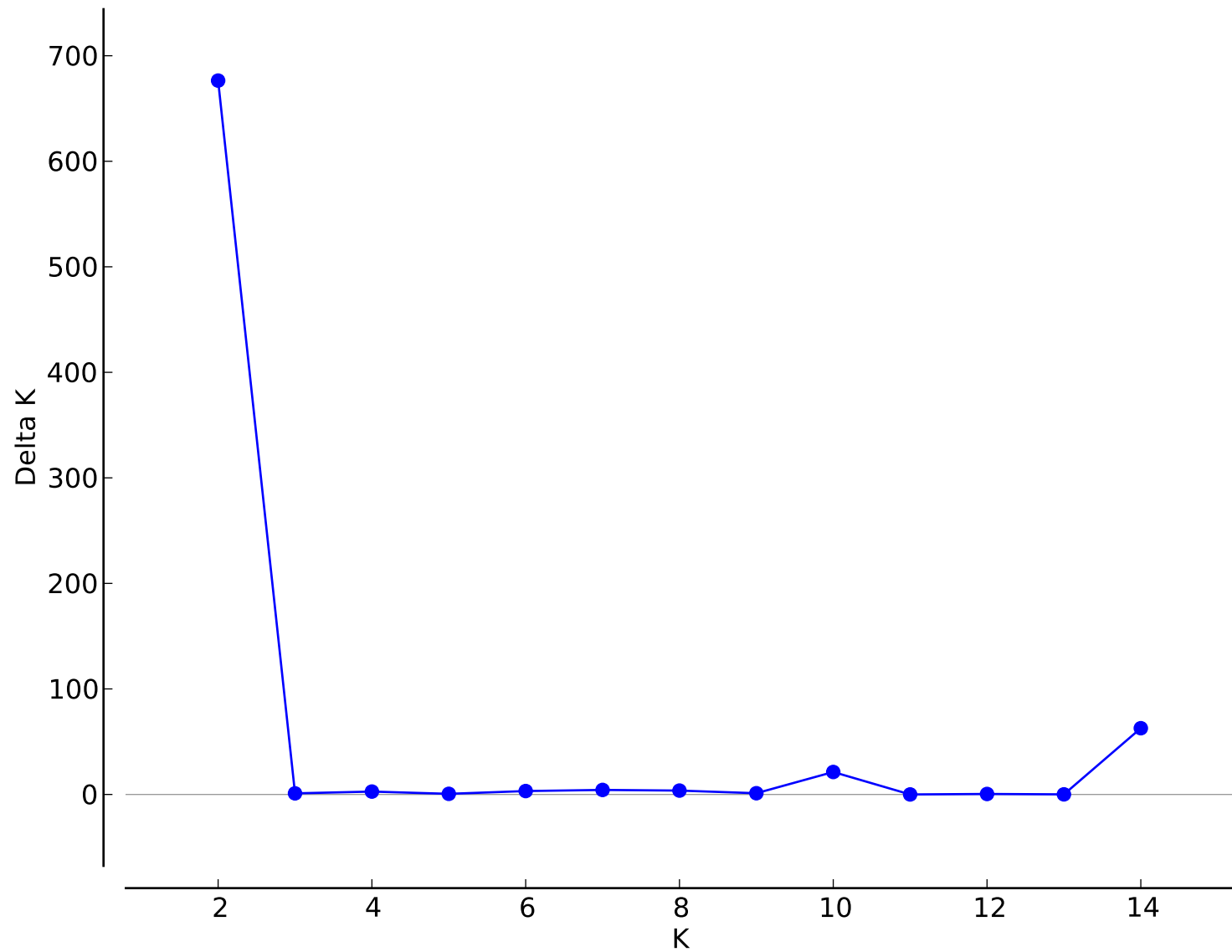

Supplement: Supplementary file 10 — Additional file 10: Figure S1. ΔK values for the Structure analysis. [file 12870_2021_2909_MOESM10_ESM.pdf]
